# The complete organelle genomes of *Crateva unilocularis* (Capparaceae)

**DOI:** 10.1186/s12863-026-01472-0

**Published:** 2026-07-19

**Authors:** Zhen-Yu Lyu, Lin Yang, Shi-Kang Shen

**Affiliations:** 1 State Key Laboratory of Vegetation Structure, Function and Construction (VegLab), Yunnan University, Kunming, 650504 China; 2 Yunnan Key Laboratory of Biological Adaptation, Conservation and Utilization, School of Ecology and Environmental Science, Yunnan University, No.2 Green lake North road Kunming, Kunming, 650504 China; 3 Ministry of Education Key Laboratory for Transboundary Ecosecurity of Southwest China, Yunnan University, Kunming, 650504 China

**Keywords:** *Crateva unilocularis*, Organelle genome, Phylogeny

## Abstract

**Objective:**

*Crateva unilocularis* is a widely consumed woody vegetable in the family *Capparaceae*. This study aimed to generate and comprehensively characterize the first complete mitochondrial genome of C. *unilocularis* using PacBio HiFi long-read sequencing, elucidating its structural organization and gene content. We also report the complete chloroplast genome for this accession. Together, these organelle genome resources provide a foundation for downstream studies on phylogeny, genome architecture, and adaptive evolution in C. *unilocularis* and related taxa.

**Data description:**

The chloroplast genome length of C. *unilocularis* is 156,525 bp, harboring 78 unique protein-coding genes (PCGs), 28 transfer RNAs (tRNAs), and four ribosomal RNAs (rRNAs). In addition, the assembly graph supports three circular mitochondrial molecules with a combined length of 566,203 bp, and junction-spanning HiFi reads validate the assembled configurations. Annotation identified 59 genes in total, including 37 PCGs, 19 tRNAs, and three rRNAs. The assembled and annotated organelle genomes represent key reference sequences for C. *unilocularis *within *Capparaceae* and will facilitate future comparative and functional genomic analyses.

## Objective

*Crateva unilocularis*, a 5–15 m tall tree in Capparaceae, is native to southern China, India, Myanmar, Laos, and adjacent regions. It holds considerable economic value: its young leaves are commonly consumed as a vegetable, and its stems are used for timber. Pharmacological studies have demonstrated that ethanol extracts of *C. unilocularis* possess hepatoprotective and glucose metabolism improving activities [1, 2]. Despite its ecological and medicinal importance, the phylogenetic relationships within *Crateva* remain unresolved, owing largely to a paucity of genomic resources. To date, only a few chloroplast genomes from *Crateva* species have been reported [3], and no mitochondrial genome has been characterized in Capparaceae, severely limiting evolutionary and adaptive trait investigations.

Organelles play a crucial role in the biological activities of living organisms, and exploring their evolutionary patterns and adaptive strategies enhances our understanding of the driving forces behind organelle differentiation [4]. Among them, chloroplast genomes are typically smaller and most exhibit a quadripartite circular structure, which makes them relatively easy to obtain. However, plant mitochondrial genomes are especially complex, they feature low substitution rates, dramatic size variation, extensive repeat content, frequent intracellular gene transfer, and abundant RNA editing sites [5–7]. In addition to the standard circular form, plant mitogenomes may adopt linear and branched configurations or multipartite structures mediated by long-repeat recombination [8]. These complexities render short‐read assemblies insufficient for obtaining complete mitogenomes, whereas PacBio HiFi long reads can span repetitive regions and resolve intricate architectures. Here, we report the chloroplast genome and the first complete mitochondrial genome of *C. unilocularis*, assembled and annotated using PacBio HiFi sequencing.

## Data description

Voucher specimens of *C. unilocularis* were collected in Dehong, Yunnan, China (102.74429°E, 25.14633°N), identified by Zhen-Yu Lyu, and deposited in the School of Ecology and Environmental Sciences, Yunnan University (Voucher Number: YDTEC002; Contact Person: Shi-Kang Shen, Email: ssk168@ynu.edu.cn). Fresh leaves were immediately flash-frozen in liquid nitrogen and stored at − 80 °C. High-molecular-weight genomic DNA was extracted using a modified CTAB protocol designed to eliminate polysaccharides and phenolic contaminants. SMRTbell libraries were prepared from the purified DNA and sequenced on the PacBio Sequel II platform. Raw long reads were assembled into contigs with Oatk v1.0 (–c 20) [9], then assembly graphs were reviewed and manually refined in Bandage [10] to resolve repetitive and ambiguous regions. Gene prediction and annotation were performed via the Plant Mitochondrial Genome Annotator (PMGA) pipeline [11] and GeSeq [12], with gene boundaries adjusted in Apollo v2.7.0 [13]. Final circular maps of each mitochondrial molecule were generated using OGDRAW [14]. To verify the correctness of the assembled structure, we extracted 1,500 bp of sequence from both sides of each junction and constructed junction-spanning reference sequences. Raw long reads were mapped back to these reference sequences using Minimap2 v2.26-r1175 [15] to assess read support across each junction. Junctions supported by at least 10 long reads spanning the corresponding junction were considered valid. The read alignments were visualized in IGV v2.17.0 [16]. To infer phylogenetic relationships within Brassicales, we extracted 33 mitochondrial protein-coding genes (PCGs) shared among 22 Brassicales species and two Malvales outgroups. Each gene was aligned separately in MAFFT [17], concatenated into a single supermatrix, and analyzed under a maximum-likelihood framework in IQ-TREE v3.0.0, using ModelFinder for substitution model selection (–m MFP) and 1,000 ultrafast bootstrap replicates (–B 1000). The resulting topology was visualized in FigTree v1.4.4.

Our sequencing dataset comprised 599,591 reads, with a total of 11,824,992,485 bases, a mean read length of 19,721.8 bp, a median read length of 19,109 bp, and a read length N50 of 20,502 bp. The chloroplast genome of *C. unilocularis* is 156,525 bp in length with a GC content of 36.3%, and exhibits the typical quadripartite structure, containing 78 unique protein-coding genes (PCGs), 28 tRNA genes, and four rRNA genes (Table 1, Data files 1–2). In addition, the complete mitochondrial genome of *C. unilocularis* was successfully assembled using long-read sequencing and exhibits a tripartite organisation comprising three distinct circular molecules (Table 1). Molecule 1 (Data file 3) spans 312,642 bp with a GC content of 44.8%, molecule 2 (Data file 4) measures 133,120 bp (GC 44.7%), and molecule 3 (Data file 5) is 120,441 bp in length (GC 44.5%). Comprehensive annotation identified a total of 59 genes (Table 1, Data file 6): 37 PCGs associated with essential respiratory, ribosomal and other mitochondrial functions, 19 tRNA genes, and three rRNA genes. The structural validation results supported the correctness of our assembled structure (Data file 7). This multipartite structure not only underscores the remarkable structural diversity of plant mitochondrial genomes but also provides a valuable reference for comparative genomics and evolutionary studies within Capparaceae. Given the absence of a published mitochondrial genome for Capparaceae, we expanded our phylogenetic framework to include 22 Brassicales species (17 genera) with two Malvales species as outgroups. Maximum-likelihood analysis resolved these Brassicales into five primary clades (Table 1, Data file 8). Three clades are represented by a single species each, while the remaining two encompass broader diversity: one comprises nine species across seven genera, and the other ten species from seven genera. *C. unilocularis* is firmly nested within Brassicales, occupying a basal position in the order. Notably, the relationships among *Brassica*, *Isatis*, and *Hirschfeldia* were not well resolved in our phylogenetic analysis.

**Table 1:** Overview of data files/data sets

Label	Name of data file/data set	File types (file extension)	Data repository and identifier (DOI or accession number)
Data file 1	The assembly and annotation chloroplast genome	Genbank file (.gb)	NCBI GenBank Database PV861827 (https://identifiers.org/ncbi/insdc:PV861827.1) [18]
Data file 2	Visualization of chloroplast genome structures. Different colors represent genes with distinct functional categories. In the inner circle, dashed segments use dark grey for GC content.	Figure (.pdf)	Figshare (https://doi.org/10.6084/m9.figshare.30683348) [19]
Data file 3	The assembly and annotation mitogenome molecule 1	Genbank file (.gb)	NCBI GenBank Database PV943379 (https://identifiers.org/ncbi/insdc:PV943379.1) [20]
Data file 4	The assembly and annotation mitogenome molecule 2	Genbank file (.gb)	NCBI GenBank Database PV943380 (https://identifiers.org/ncbi/insdc:PV943380.1) [21]
Data file 5	The assembly and annotation mitogenome molecule 3	Genbank file (.gb)	NCBI GenBank Database PV943381 (https://identifiers.org/ncbi/insdc:PV943381.1) [22]
Data file 6	Visualization of mitochondria genome structures. A, Recombination structure of the mitochondrial genome. B, Genes inside and outside the circle are transcribed counterclockwise and clockwise, respectively. Different colors represent genes with distinct functional categories. In the inner circle, dashed segments use dark grey for GC content.	Figure (.pdf)	Figshare (https://doi.org/10.6084/m9.figshare.30683348) [19]
Data file 7	Long read validation of the junction in mitochondrial molecule 1. Raw long reads continuously span the junction-spanning reference sequence, supporting the assembly of the eight junctions. The grey bars represent individual long reads.	Figure (.pdf)	Figshare (https://doi.org/10.6084/m9.figshare.30683348) [19]
Data file 8	Maximum likelihood phylogenetic tree inferred from concatenated mitochondrial protein-coding genes of *C. unilocularis* and representative Brassicales species. Bootstrap support values are shown above the branches. The inset in the upper left shows the same phylogenetic tree with branch length information.	Figure (.pdf)	Figshare (https://doi.org/10.6084/m9.figshare.30683348) [19]
Data set 1	PacBio HiFi raw data	fastq files (.fq)	NCBI Sequence Read Archive SRR36135288 (http://identifiers.org/ncbi/insdc.sra:SRR36135288) [23]

## Limitations

The mitochondrial and chloroplast genomes were assembled from a single *C. unilocularis* accession, which limits our ability to assess intraspecific variation, population structure, and potential organelle genome polymorphisms within the species.

## Data Availability

The sequence data supporting the findings of this study are openly available in NCBI GenBank under the accession No. PV943379-PV943381 and PV861827. The associated BioProject, SRA, and BioSample numbers are PRJNA1367767, SRR36135288, and SAMN53352702, respectively. All assembly and annotation files can also be obtained at figshare (https://doi.org/10.6084/m9.figshare.30683348).
